# Sub-Lethal Doses of Clothianidin Inhibit the Conditioning and Biosensory Abilities of the Western Honeybee *Apis mellifera*

**DOI:** 10.3390/insects10100340

**Published:** 2019-10-11

**Authors:** Merle T. Bartling, Andreas Vilcinskas, Kwang-Zin Lee

**Affiliations:** 1 Institute for Insect Biotechnology, Justus-Liebig-University, Heinrich-Buff-Ring 26-32, D-35392 Giessen, Germany; merle.t.bartling@agrar.uni-giessen.de (M.T.B.); andreas.vilcinskas@agrar.uni-giessen.de (A.V.); 2 Fraunhofer Institute for Molecular Biology and Applied Ecology, Winchester Strasse 2, D-35394 Giessen, Germany

**Keywords:** insecticide, conditioning, neonicotinoid, APIS chamber, aversion test

## Abstract

Insects play an important role in the stability of ecosystems by fulfilling key functions such as pollination and nutrient cycling, as well as acting as prey for amphibians, reptiles, birds and mammals. The global decline of insects is therefore a cause for concern, and the role of chemical pesticides must be examined carefully. The lethal effects of insecticides are well understood, but sub-lethal concentrations have not been studied in sufficient detail. We therefore used the western honeybee *Apis mellifera* as a model to test the effect of the neonicotinoid insecticide clothianidin on the movement, biosensory abilities and odor-dependent conditioning of insects, titrating from lethal to sub-lethal doses. Bees treated with sub-lethal doses showed no significant movement impairment compared to untreated control bees, but their ability to react to an aversive stimulus was inhibited. These results show that clothianidin is not only highly toxic to honeybees, but can, at lower doses, also disrupt the biosensory capabilities of survivors, probably reducing fitness at the individual level. In our study, sub-lethal doses of clothianidin altered the biosensory abilities of the honeybee; possible consequences at the colony level are discussed.

## 1. Introduction

Insects are an integral part of many ecosystems, acting as prey for larger animals, and fulfilling important functions, such as pollination and nutrient cycling. The abundance of flying insects in nature reserves across Germany has declined by 75% over the past 25 years [1]. The cause of this massive decline is unclear, although possibilities include climate change, habitat destruction and the widespread use of agrochemicals [2]. Many agricultural monocultures do not thrive without the application of chemical fungicides, herbicides and insecticides to reduce yield losses [3]. Approximately 2.8 million tons of insecticides are used worldwide every year [4], including synthetic neonicotinoids, which were first used as seed dressing in the late 1990s [5]. Because of their systemic mode of action, neonicotinoids pass from the treated seed into the complete growing plant, and thus also into the nectar and pollen [6]. Many bee species are currently threatened by different factors, such as habitat loss, climate change and stressors, like exposure to insecticides [2,7]. This exacerbates the impact of other factors, and leads to high casualty rates among domesticated species such as the western honeybee *Apis mellifera*, and wild species such as *Bombus terricola* and *B. occidentalis*, which were nearly exterminated in the late 1990s [2]. Whereas for managed honeybees working solutions has been developed [8,9,10], wild bees and other pollinators are still under considerable threat.

Although the direct lethal effect of insecticides upon insects is clear, the impact of sub-lethal concentrations can have many different effects, as has been conducted under field-realistic conditions, with some opposing results [11,12,13,14,15,16,17]. Sub-lethal doses of neonicotinoids can affect insect memory and recall [18,19,20,21]. For example, imidacloprid interferes with learning behavior, olfactory orientation and flight activity in honeybees, even when low doses of 100, 500 and 1000 ppb are added to food, whereas no mortality could be observed for the high dose of 1000 ppb (one part per million) [22]. Of note, there is considerable variability in the LD50 values of imidacloprid between different studies [23]. Furthermore, most bees fed on diets supplemented with sub-lethal doses of thiamethoxam corresponding to a real dose of 1.34 ng in a 20 µL sucrose solution, were unable to find their way back to the hive [24]. Honeybees that do not return from foraging (or misrepresent the location of food sources) reduce colony fitness [25,26,27].

Previous studies of neonicotinoids have considered the effects of imidacloprid, thiamethoxam and clothianidin, the latter with high toxic properties causing the Rheintal incident, upper Rhine valley, Germany, in 2008 [28]. To this end, we selected the western honeybee as a model to test the potential sub-lethal effects of clothianidin. Western honeybee workers undertake a variety of tasks during their lifetime [29,30] and the organization of tasks inside the colony is determined by the age of the workers and feedback from the hive in order to respond to the hive’s needs [31]. The ability to communicate information about the odor and position of nectar-rich flowers is learned when the workers (female) become foraging bees, and the learning process is continuous [32].

Here we used the automatic performance index system (APIS), a conditioning chamber based on aversive stimuli, to test the conditioning ability of bees exposed to different doses of clothianidin in their food [33,34]. Using this experimental setup, we addressed the hypothesis that bees exposed to sub-lethal doses of clothianidin differ from unexposed controls in their ability to react when presented with odor cues. This would suggest that, in addition to the lethal effect of high-dose clothianidin exposure, lower concentrations may disrupt learning and memory. Furthermore, we offer with the APIS chamber a standardized approach to measure these sub-lethal effects of pesticides at the individual bee level to gain valuable insights for the understanding of the role of pesticides in sub-lethal concentrations in honeybee toxicology.

## 2. Materials and Methods

### 2.1. Honeybees

Western honeybees were collected from a hive at the Fraunhofer Institute for Molecular Biology and Applied Ecology, Giessen, Germany (50°34′05.8″ N, 8°40′18.6″ E). Honeybees departing from the hive were taken from May 2016 until September 2016 from the hive entrance and were stored before treatment in small groups of 20–30 bees within ventilated plastic boxes (10 × 10 × 8 cm). The storage boxes were equipped with a drinking water source and a feeding station containing Apiinvert™ sugar syrup solution (Südzucker AG, Mannheim, Germany). The sugar syrup consisted of sucrose (310 mg/g), glucose (300 mg/g) and fructose (390 mg/g), and had a feed value of one kilogram of crystalline sugar per liter of solution. The honeybees were stored in the dark at room temperature prior to the feeding.

### 2.2. Treatments

For the clothianidin experiments, two different stock solutions were prepared and fed to the bees over a period of 24 h. The control diet consisted of 99% Apiinvert™ sugar syrup and 1% deionized water. The clothianidin-spiked experimental diet consisted of 99% Apiinvert™ sugar syrup supplemented with 100 μL/L clothianidin (Bayer CropScience AG, Monheim am Rhein, supplied by the Bee Institute Kirchhain) at different final concentrations, which were diluted in deionized water (Table 1). The stock solutions were stored at −20 °C, and dilutions of the clothianidin-spiked diet were prepared by mixing with Apiinvert™ shortly before use, resulting in one control diet and three experimental diets comprising a 10-fold dilution series of clothianidin (Table 1). Before use, every diet was vigorously stirred.

The control group was fed with 70 µL of control diet per bee, whereas the treatment group was fed with 70 µL Apiinvert™ per bee, spiked with three different clothianidin concentrations (see Table 1). No additional water source was presented. The fed bees were kept individually in 50 mL reaction tubes (Falcon, Cole-Parmer, Vernon Hills, IL, USA) in a dark climatic chamber at 26 °C and 60% humidity.

Honeybees were randomly assigned to one of the four diet groups and were allowed to access the syrup for 24 h. During this time, the fed animals were placed individually in separate tubes and maintained at 26 °C and 60% humidity in a dark climatic chamber. After 24 h, we recorded the number of dead bees in each diet group, and 45 survivors from each group were tested in the automatic performance index system (APIS) conditioning chamber. Each honeybee represented one biological replicate of the test, because it was necessary to condition every bee individually. Afterwards, the clothianidin-fed bees were stored at −80 °C, whereas the control bees were color marked on the thorax before releasing them to their hive. In rare cases where marked bees were recaptured, they were not used in subsequent experiments to ensure that all conditioned bees were initially naïve.

### 2.3. Movement Assay

The movement assay [35] was modified as follows, with 30 bees assigned to each group. The control group was fed with 70 µL of control diet per bee, whereas the treatment group was fed with 70 µL of 42.86 pg/µL (corresponding to 42.86 ppb) clothianidin in pure Apiinvert™ per bee, the highest used concentration for this study. No additional water source was presented. The fed bees were kept individually in a dark climatic chamber at 26 °C and 60% humidity. After a feeding period of 24 h, the bees were observed for 5 min one after another in a Petri dish (Greiner Bio-One, Kremsmünster, Austria). The dish was divided into four sections of equal size with markings. The intensity of movement was measured by counting how often each bee crossed a boundary line.

### 2.4. Conditioning

The APIS chamber (148 mm long, 20 mm wide and 6 mm deep) was constructed from acrylic glass (Makrolon, Bayer MaterialScience AG, Leverkusen, Germany). It was a closed system with an electrode-covered interior into which various odors could be introduced [36]. In order to prevent the distraction of the bee by optical stimuli, the chamber was surrounded by a LEGO™ platform including a lid. Different odors could be introduced at each end, at a flow rate of 90 ± 0.1 mL/min, via flexible tubes connected to a pumping system. The two halves of the system were separated by a stream of air flowing at 50 mL/min to ensure that only one half of the chamber was flooded with the released odor. The chamber was equipped with light barriers to detect the location of the bee, and the odorant was pumped into the side where the bee was located.

A 1% solution of 1-hexanol or 1-decanol (both Sigma-Aldrich, Munich, Germany) in mineral oil was used on each side of the chamber for the prefrontal syringes, with pure mineral oil as a blank for the posterior. 

Then, 150 μL of test substance was pipetted onto a Sugi suction strip (Kettenbach GmbH & Co. KG, Eschenburg, Germany), which was rolled up and placed in a 2-mL disposable syringe (B. Braun Melsungen AG, Melsungen, Germany). Single-use cannulas (B. Braun Melsungen AG) were used to connect the flexible tubes and the syringe.

Aversive conditioning was achieved by applying a mild electric shock on 1-hexanol or 1-decanol depending on the protocol selected, thus ensuring that neither of the two odors was favored from the outset by the honeybee. The odor was introduced for 8 s into the side of the chamber where 26 infrared LED sensors recognized the bee. During the training phase, the bee received a weak electric shock (10 V, 1.2 Hz, pulse duration 200 ms) after the first 2 s, when the aversively conditioned scent was introduced, and this stopped when the bee crossed the middle of the chamber.

As an example of the training process, the bee was confronted during the training phase with the punished odor A, known as the conditioned fear stimulus (CS+), and the unpunished odor B, known as the conditioned safety stimulus (CS−), in the order ABBABAAB. The inter-trial intervals were set to 34 s. After a rest period of 5 min, the learned behavior was interrogated without electric shocks in the sequence ABBA. The aversively-conditioned odor, and the order in which the training occurred and the learned knowledge was queried, and then varied with the eight different protocols to test the two presented odors in all possible orders.

To monitor the behavior of the honeybee inside the chamber, movement was recorded continuously using 26 infrared LED sensors via an automatic tracking program. The success of the training was reported as the attractance index (AI), based on the running path of the bee when the odor was introduced, and was calculated according to the reaction of the bee. The AI is defined as the area of a peak generated between the opening and closing of the valves.

### 2.5. Statistical Analysis

For the movement assay, we determined differences between the control and treatment groups using a paired t-test after checking for a normal data distribution. For the APIS chamber experiments, data analysis was carried out using R i368 v3.2.2 (The R Foundation for Statistical Computing, Vienna, Austria), Microsoft Excel 2010 (Microsoft Corporation, Redmond, USA) and RStudio (RStudio, Boston, USA). Diagrams were generated using GraphPad Prism (GraphPad Software, Inc. La Jolla, CA, USA). The text files created in APIS were converted to Excel files using an R script prepared by Nicholas Kirkerud (Institute of Neurobiology, University of Konstanz, Germany) and adapted by Matthias Schott (Aquatic Chemical Ecology working group, University of Cologne, Germany). The resulting file was modified in Microsoft Excel. Statistical analysis was performed using RStudio and GraphPad Prism. A Shapiro-Wilk test was applied to test the data for normal distribution, based on the difference in mean AI values for the non-punished and punished odors. If a normal distribution was confirmed, a paired t-test was carried out with the average movement (see movement experiments) or AI values of the CS+ and CS− conditions (aversion training) of all the bees to test for differences between the two odors.

## 3. Results

### 3.1. Mortality Rates in the Treatment and Control Groups

**Figure 1:**
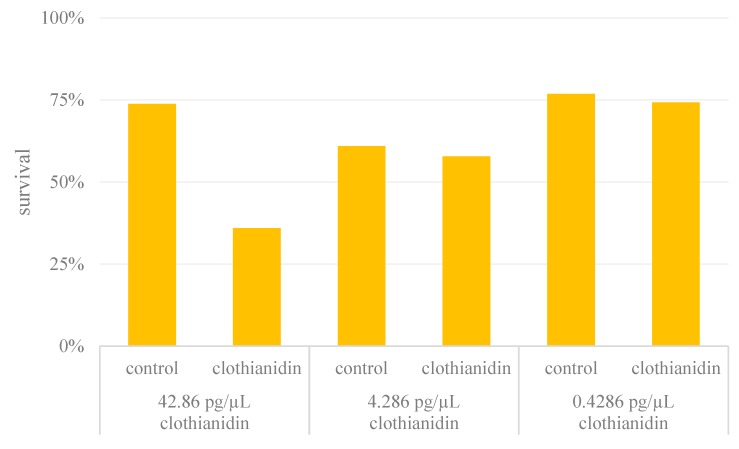
Mortality rates expressed as the proportion of living bees in the pesticide and control groups. For each concentration of clothianidin, a separate control group was established.

We captured 596 forager bees in total for the feeding experiments, among which 382 survived the 24-h exposure period and were tested in the APIS chamber. The mortality rate among the bees fed on the control diet was ~26%, with some variation in the different experiments. In contrast, the mortality of the bees in the clothianidin treatment groups was strictly dose-dependent, with 64% mortality at a dose of 3000 pg per bee, falling to 42% at 300 pg per bee and 26% at 30 pg per bee (Figure 1).

Therefore, more bees were needed in the clothianidin-treatment groups to ensure that a near equal number of treated and control bees survived for experiments in the APIS chamber (Table 2). All the data satisfied the Shapiro-Wilk test for normal distribution (*p* > 0.05).

### 3.2. Movement Impairment in the Treatment and Control Groups

**Figure 2:**
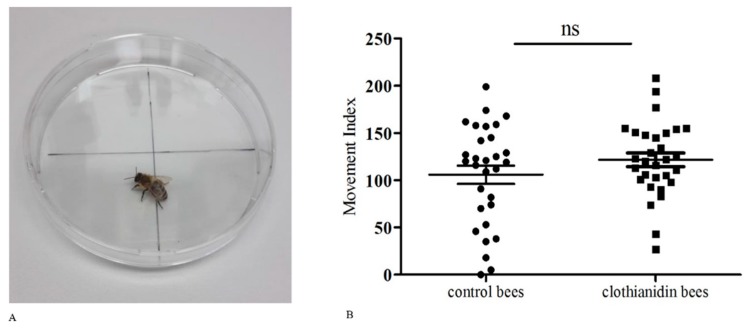
Movement assay. (**A**) Experimental setup for the movement assay, in which we observed bee movement between the four sections of a Petri dish. (**B**) Movement index of control and clothianidin-fed bees expressed as the frequency of crossing the different quadrants, each point representing the movement index of one bee. For statistical analysis we used a paired t-test to test for differences between the groups (*p* = 0.2745; *n* = 30 honeybees per group; ns = non-significant). The mean value and the standard error of the mean (SEM) are also shown.

To determine whether the bees were already impaired in terms of movement following treatment with sub-lethal doses of clothianidin, we compared the intensity of movement in the control and clothianidin treatment groups (Figure 2) and observed no significant differences in the movement index (paired *t* test, *p* = 0.2745, *n* = 30 for each group). We therefore concluded that sub-lethal doses of clothianidin had no significant effect on honeybee movement, although this experiment cannot rule out the possibility that more subtle effects on movement are caused by clothianidin. All data were tested for normal distribution (Shapiro-Wilk normally test, control bees *p* = 0.1866; clothianidin bees *p* = 0.8004).

### 3.3. Conditioning and Biosensory Abilities in the Treatment and Control Groups

**Figure 3:**
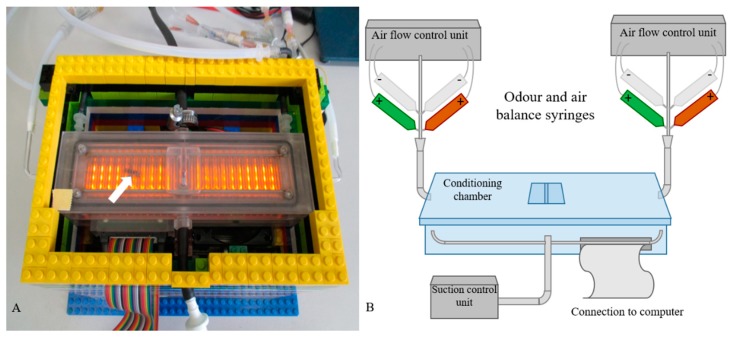
The automatic performance index system (APIS). (**A**) Photograph of an APIS conditioning chamber with a honeybee inside (white arrow). The orange light was activated to visualize the bee and the electrodes inside the chamber. The bee was introduced through the entrance in the middle. The lateral hoses introduce the two odors, whereas those in the middle separate the chamber into halves by introducing air flow. The LEGO™ platform is cooled by fans, and the chamber is connected to a computer. (**B**) Schematic representation of the experimental setup, modified from the original reported version [33]. The syringes marked (+) are the odor and air balance syringes (odors: green = 1% 1-hexanol in mineral oil, red = 1% 1-decanol in mineral oil). The syringes marked (–) contain mineral oil as a blank.

The surviving bees were tested in the APIS conditioning chamber [33,36] (Figure 3) and we conditioned the bees with two different odors: 1-hexanol and 1-decanol [37].

**Figure 4:**
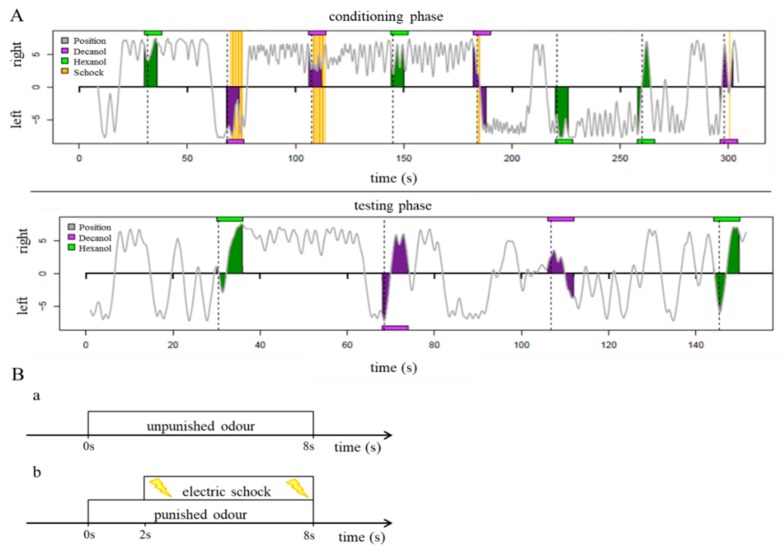
The conditioning process. (**A**) Recording the conditioning and test phases in the APIS conditioning chamber. The gray line describes the path of the bee between the left end (−7.4 cm) and right end (+7.4 cm) of the chamber. The colored boxes mark the introduction of the two odors. The yellow mark indicates the delivery of an electric shock. (**B**) The unpunished odor is introduced to the side of the chamber containing the bee for 8 s without further stimulus (**a**), whereas the punished odor is introduced also for 8 s, but a weak electric shock is delivered after 2 s via electrodes on the side of the chamber containing the bee (**b**).

**Figure 5:**
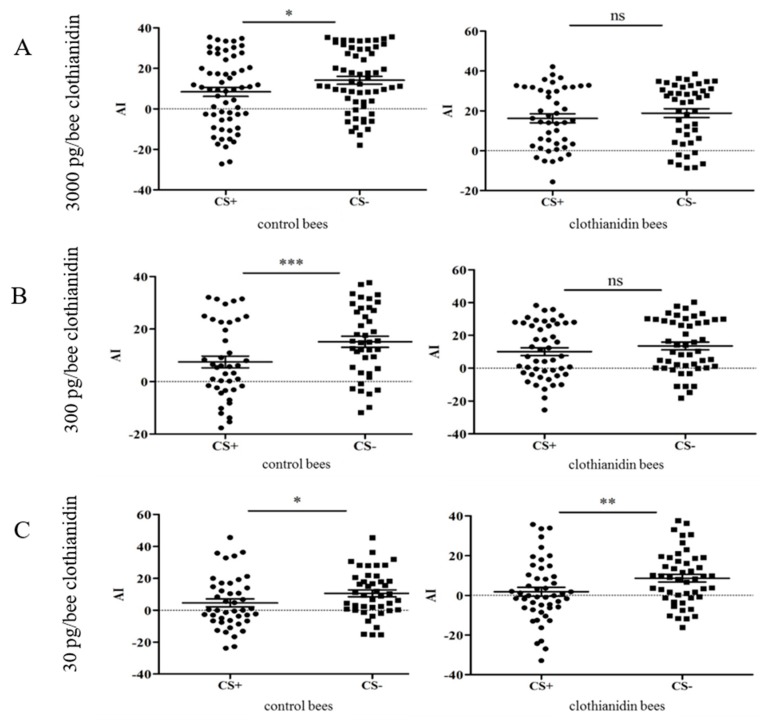
Attractance index (AI) for the punished odor (CS+) and the non-punished odor (CS−) in treatments with different concentrations of clothianidin. (**A**) At a clothianidin dose of 3000 pg per bee, the control bees were able to distinguish between CS+ and CS−, but those exposed to clothianidin were not (control, *p* = 0.019, *n* = 59; 3000 pg clothianidin per bee, *p* = 0.154, *n* = 45). (**B**) Similar results were observed when the dose was 300 pg clothianidin per bee (control, *p* = 0.0009, *n* = 41; 300 pg clothianidin per bee, *p* = 0.112, *n* = 48). (**C**) In the experiment with 30 pg clothianidin per bee, both the control and clothianidin groups were able to distinguish between CS+ and CS− (control, *p* = 0.0144, *n* = 43; 30 pg per bee clothianidin, *p* = 0.0048, *n* = 49). On each graph, each bee is represented by one point for CS+ and one for CS−. For statistical analysis we used a paired t-test to test for differences between the groups. The mean value and the standard error of the mean (SEM) are also shown.

One of the odors was presented with an aversive stimulus (Figure 4). The AI values of the bees in the clothianidin treatment groups were compared to those of the control bees (Figure 5). At a high dose of clothianidin (3000 pg/70 µL per bee), we observed a clear difference in behavior between the treatment and control groups (Figure 5A). Whereas bees fed on the control diet could easily distinguish between the CS+ and CS− conditions and showed avoidance behavior towards the punished odor (paired t-test, *p* = 0.0191; Shapiro-Wilk test, *p* = 0.1731; *n* = 59), the clothianidin-fed bees could not make this distinction (paired t-test, *p* = 0.154; Shapiro-Wilk test, *p* = 0.151; *n* = 45). At a dose of 300 pg clothianidin per bee, we likewise observed a difference in behavior between the treatment and control groups (Figure 5B). Again, the control bees could distinguish between the CS+ and CS− conditions and showed avoidance behavior during the test phase (paired *t* test, *p* = 0.0009; Shapiro-Wilk test, *p* = 0.899; *n* = 41), but the clothianidin-fed bees lacked this ability, and showed no avoidance behavior in the presence of the punished odor (paired *t*-test, *p* = 0.112; Shapiro-Wilk test, *p* = 0.8722; *n* = 48).

In contrast to the higher doses described above, there was no significant difference in behavior between the treatment and control groups at a dose of 30 pg clothianidin per bee: Where *p* = 0.0144 for the control bees (paired *t*-test; Shapiro-Wilk test, *p* = 0.2883; *n* = 43), and *p* = 0.0049 for the clothianidin-fed bees (paired *t*-test; Shapiro-Wilk test, *p* = 0.2263; *n* = 49). The bees in both treatments were therefore conditioned to associate the punishment with an odor, and to avoid it during the test phase (Figure 5C).

## 4. Discussion

Neonicotinoid insecticides are lethal to insects by design, but even sub-lethal doses could lead to a range of physiological and behavioral effects that have a deleterious impact on beneficial insects such as bees. In order to investigate these effects, we captured almost 600 honeybees and fed them on diets spiked with three different concentrations of clothianidin, or on a control diet without the insecticide. The dose of a substance that is lethal to 50% of the tested population is defined as the LD50 [38]. The LD50 of clothianidin taken orally is 3790 pg per bee [39], according to the manufacturer Bayer CropScience, whereas the LD50 on surface contact is 44 ng per bee [40]. The highest dose we used in our experiments was 3000 pg per bee, which is close to the LD50 value. This explains the observed mortality of 64% in the treatment group, part of which is due to the insecticide, and part due to the stress of captivity (the latter resulting in 26% mortality even in the control group).

Another factor might be the sampling of bees, where we cannot exclude differences in the age composition that could lead to the high mortality [41]. In contrast to earlier studies, we treated single honeybees so that each of them can be treated as an independent experimental unit [35], whereas most other studies housed at least 30 individuals per cage [42,43]. Bearing in mind that honeybees are social animals, this may explain the elevated baseline of lethality under control conditions. However, when keeping honeybees in the laboratory, we used conditions recommended for the long-term maintenance of adult honeybees for laboratory experiments, i.e., a 3:1 ratio of the cage volume in cm^3^ to the number of bees [44]. The selection of surviving bees after the clothianidin treatment poses also the possibility that the selection is towards those bees that did not fully intake the clothianidin. For further studies, we suggest a quantification of the food intake of each individual bee to exclude this potential deviation.

Our results indicate that 30–300 pg per bee represents a toxicity threshold for clothianidin in this species. The short exposure time of 24 h and the number of dead animals also gives an impression of the strong impact of clothianidin on the individual level. It takes 21 days for a fertilized egg to become a worker. One hypothesis is that, due to the long development time, the number of fully developed female workers cannot compensate for the loss caused by poisoned bees, resulting in a shortage of foraged food, and therefore a weakened hive [24,45]. One caveat, though, is that effects observed under laboratory conditions are often not detected under field realistic studies [12,13,14,15,16,17,46,47,48], and the underlying mechanism of the patterns and processes of pesticide exposure on individuals and their transmission to colony level effects are not well understood. The toxic effects for sub-lethal doses of a neonicotinoid, as presented here under laboratory conditions, has therefore direct consequences on an individual level, and resulting physiological effects could have an impact on the homing of individual honeybees [24]. But if enough foragers suffer from homing failure, colony-level functions could be impaired, such as food acquisition and brood rearing. At the doses we used, clothianidin had no significant effect on the locomotion of the bees. Because the APIS chamber test relies on the movement of conditioned bees, it was important to rule out any substantial negative effects of clothianidin on movement. The most important outcome of our experiments was that clothianidin showed a significant and dose-dependent effect on the conditioning ability of honeybees. Thus, when the experimental groups were exposed to 3000 pg or 300 pg of clothianidin per bee, aversive conditioning behavior was inhibited, and only the control group was able to show avoidance behavior. These results indicate that clothianidin disrupts the olfactory conditioning process or the retrieval of information at doses of 300 pg and especially 3000 pg per bee, but at 30 pg per bee (~100 times lower than the 24-h LD50) the effect of the insecticide on conditioning was not statistically significant.

Olfactory conditioning is also disrupted by other neonicotinoids. Bees fed on a sub-lethal concentration of the related neonicotinoid imidacloprid showed a significantly more limited proboscis extension response in classical scent conditioning experiments, where the presentation of an odor is followed by rewarding the bees with a sugar solution [19,26,27]. When the odor is offered to conditioned animals a second time, they stretch out their proboscis in anticipation of the reward. Bees exposed to imidacloprid showed a statistically significant reduction in this behavior. Our results indicate that clothianidin similarly alters the behavior of honeybees in the presence of olfactory stimuli.

The effect of clothianidin on the orientation ability of bees has been tested, revealing that the exposed bees select a significantly longer and more time-consuming homeward journey [21]. Sensomotor effects were not observed, i.e., the homeward flight path (vector flight) was undisturbed. This flight can be observed even if bees are taken away from a feeding place and released at another location, even though it does not lead the bees to their home. The prerequisite of a vector flight is the orientation flight. Young bees in particular fly away from the area of the hive in order to orient themselves, later using landmarks. The results discussed above indicate that a sub-lethal dose of neonicotinoids interferes with spatial memory, or the retrieval of that memory. Clothianidin-induced disorders are permanent and irreversible [45]. 

The effects of neonicotinoids are even more potent in bee larvae, where exposure to as little as 40 pg imidacloprid per larva inhibits learning behavior at the adult stage [49].

Workers display complex social behaviors that are often regulated by olfactory signals and communication via pheromones [50], and the search for nectar and pollen is also controlled by their sense of smell [51]. The effectiveness of a forager is thus determined by her olfactory abilities, and the presence of insecticides that inhibit olfactory learning and memory can negatively affect the overall health of the colony. Imidacloprid reduces the intensity and frequency of the waggle dance when foragers return to the hive [52]. The misrepresentation of information about food sources limits the effective recruitment of other foragers, and thus causes the weakening of the colony [53].

In Germany and throughout the EU, the application of clothianidin has been banned for all outdoor uses since September 2018. However, studies have shown that neither declines of honeybee colonies, nor of wild pollinators, increased during the time when neonicotinoids were admitted [54,55]. Where use is permitted, clothianidin is applied as a seed coating, and is therefore absorbed easily from the soil, transported to all plant tissues via the xylem, and ultimately enriched in the pollen and nectar [6]. It also spreads to areas surrounding the treated field, and thus reaches non-target plants and wildflowers visited by bees [56,57,58]. Clothianidin is very stable in soil with a half-life of 148–1155 days, and is therefore likely to accumulate in soil due to successive applications in different seasons [59]. The levels of clothianidin we tested in this study were higher than the 2.24–5.7 ng/g previously reported as field concentrations in nectar and pollen [60,61,62,63]. However, bees may encounter the insecticide via several alternative routes, including the guttation water released by plants. This water is used for the thermoregulation of the hive but also for the production of the brood feed [64,65]. The quantity of clothianidin residues found in guttation water decreases over time [66] but can still be detected up to one month after the first sampling [46]. However, there are other studies indicating that field realistic concentrations of neonicotinoid residues are either overestimated [46], or show no adverse effect on honeybees at all [63,67], exemplifying the difficulty to obtain solid residue value limits or their implication in the field. Therefore, it is important to understand the mechanisms underlying sub-lethal effects on an individual level, and build up from there to the colony level. So far, an easy and reliable tool to obtain quantifiable data on the sub-lethal effects of pesticide exposure on the individual level was missing. We present here the use of the APIS chamber that provides a standardized operational method giving valuable insights to this topic. The APIS chamber is a qualitative and quantitative read-out system that could also be applied in the regulatory risk assessment of plant protection products.

## 5. Conclusions

Our study on honeybees under laboratory conditions confirmed the lethality of clothianidin to honeybees at high doses. We could as well show that individual honeybees fed with sub-lethal doses (30 pg to 3000 pg per bee) revealed inhibitory effects on conditioning responses using the APIS chamber. The use of the APIS chamber in the context of studying these sub-lethal effects of pesticides is to our knowledge the first time to be described, and offers a reliable tool to assess the condition capability of treated honeybees. In perspective, the APIS chamber could be used in the regulatory assessment of plant protection products.

## Figures and Tables

**Table 1:** Nominal values of clothianidin in the diets per microliter and per bee. The food was given over 24 h to single captured bees, providing it to the bees in 1.5 mL reaction tube caps (Eppendorf AG, Hamburg, Germany) as feeding bowls.

Diet	Amount of Clothianidin per Microliter Sugar Syrup	Amount of Clothianidin per Bee/70 µL
Control	0 pg	0 pg
High	42.86 pg	3000 pg
Medium	4.286 pg	300 pg
Low	0.4286 pg	30 pg

**Table 2:** Listing of the experimental bees of the clothianidin test. Shown is the number of fed control and clothianidin bees of the three sampled dilution steps and the number of experimental bees sampled after 24 h of exposure to the food in the automatic performance index system (APIS) conditioning chamber.

	Control	3000 pg/Bee Clothianidin	300 pg/Bee Clothianidin	30 pg/Bee Clothianidin
fed bees	80	128	83	66
experimental bees	59	45	41	48
